# Extracellular vesicle: A magic lamp to treat skin aging, refractory wound, and pigmented dermatosis?

**DOI:** 10.3389/fbioe.2022.1043320

**Published:** 2022-11-07

**Authors:** Haiyan Wu, Zhenchun Zhang, Yuemeng Zhang, Zhenlin Zhao, Hongming Zhu, Changwu Yue

**Affiliations:** ^1^ Key Laboratory of Microbial Drugs Innovation and Transformation of Yan’an, School of Basic Medicine, Yan’an University, Yan’an, China; ^2^ Institute for Regenerative Medicine & Research Center for Translational Medicine, Shanghai East Hospital, Tongji University School of Medicine, Shanghai, China; ^3^ Shenzhen Ruipuxun Academy for Stem Cell & Regenerative Medicine, Shenzhen, China

**Keywords:** skin aging, pigmented dermatosis, refractory wound, extracellular vesicles, therapeutic applications, engineering exosomes, medical aesthetics

## Abstract

Exposure of the skin to an external stimulus may lead to a series of irreversible dysfunctions, such as skin aging, refractory wounds, and pigmented dermatosis. Nowadays, many cutaneous treatments have failed to strike a balance between cosmetic needs and medical recovery. Extracellular vesicles (EVs) are one of the most promising therapeutic tools. EVs are cell-derived nanoparticles that can carry a variety of cargoes, such as nucleic acids, lipids, and proteins. They also have the ability to communicate with neighboring or distant cells. A growing body of evidence suggests that EVs play a significant role in skin repair. We summarize the current findings of EV therapy in skin aging, refractory wound, and pigmented dermatosis and also describe the novel engineering strategies for optimizing EV function and therapeutic outcomes.

## Introduction

As the largest barrier organ, which is constantly exposed to the external environment, skin has a relatively high risk of suffering injuries due to genetic make-up, lifestyle, nutrition, solar radiation, and other environmental factors (Hong et al., 2021; Meccariello and D'Angelo, 2021). Aging and diseases of skin pose a multidimensional burden that includes mental, social, and financial consequences for patients, families, and society. Although skin abnormalities have attracted considerable attention worldwide for decades, available treatments have not achieved desirable effects and need to be further studied (Ogawa, 2017; Heidari Beigvand et al., 2020).

EVs are nanosized vesicles with phospholipid bilayer membranes. They can carry important gene information to recipient cells, such as lipids, proteins, carbohydrates, and nucleic acids (e.g., DNA, mRNA, miRNA and lncRNA, etc.) (Hong et al., 2019a). They can generally regulate biological events (e.g., cell proliferation, differentiation, apoptosis, migration, and immunomodulatory reactions) in recipient cells by conveying their inclusions. According to their diameter, EVs can be classified into six subpopulations: exomeres, with a diameter of less than 50 nm; exosomes, with a diameter of 30–150 nm; Ectosomes or shedding microvesicles, with a diameter of 100–1000 nm; apoptotic bodies, with a diameter of 1000–5000 nm; migrasomes, with a diameter of 500–3000 nm; and large oncosomes, with a diameter of 1000–10,000 nm (Anand et al., 2021).

EVs are secreted from almost all cell types as vehicles of intercellular communication and information transfer (Hong et al., 2019b). The vital role of EVs in tissue homeostasis and repair has recently been demonstrated (Kalluri and LeBleu, 2020; Sun et al., 2021), but our understanding of their function and mechanism in skin dysfunction and diseases is still in its infancy (Wang et al., 2019b; Guo et al., 2021).

In this review, we summarize the recent work on the functions of EVs in severe skin problems (i.e., aging, refractory wounds, and pigmented dermatosis) and outline the strategy of engineered modifications of EVs in skin therapy. Based on these advances, we discuss the current challenges and future perspectives.

### EVs improve skin aging

Exogenous factors (e.g., sunlight, ionizing radiation, pollution, toxins, and other factors) can cause the appearance of skin discoloration, sagging and dullness, roughness, deep wrinkles, and even loss of skin elasticity, of which photoaging is the most common (Meccariello and D'Angelo, 2021). Endogenous senescence is also called inherent aging and is affected by endocrine and genetic factors and reflects a degradation process of the entire organism (Del Bino et al., 2018). Stem cells have the capacity for excellent regeneration and growth and have gained great interest in the scientific community since their discovery by Till and McCulloch in 1961. They are widely used in the treatment of various diseases because of their capacity to differentiate into various cell types of the body, including limb ischemia, organ failure, skin wound, and cartilage defects (Goradel et al., 2018; Alessandrini et al., 2019; Ryu et al., 2020; Nammian et al., 2021). However, potential risks and technical challenges continue to trouble the clinical application of stem cells, including tumorigenicity, transplant rejection, differentiation and storage efficacy, and ethical issues (Gonzalez Villarreal et al., 2018; Volarevic et al., 2018). Recently, stem cell-secreted EVs, carrying a wide variety of RNA and proteins, have been considered one of the most attractive cell-free treatment modalities in the regenerative medicine field (Whiteside, 2018; Wu et al., 2018).

The effects of stem cell-derived EVs on skin aging and aging-related disorders have been proven (Saxena and Kumar, 2020). Dorronsoro et al. (2021) Akaitz et al. investigated the anti-aging property of EVs driven from mesenchymal stem cells (MSCs). They found that EVs released by MSCs, which derived from human embryonic stem cells and bone marrow, significantly decrease the expression of senescence markers (p16, p21, IL-6 and IL-1β) and improve health span in senescent fibroblasts, naturally aged wild-type and Ercc1^−/∆^ mice (a model of premature aging) *in vivo*. Shi et al. (2021b) found that gingiva MSC-derived EVs downregulate the expression of senescence-related genes including *ß*-galactosidase, p53, p21, and γH2AX and the mammalian target of rapamycin (mTOR)/pS6 signaling pathway in aging human skin fibroblasts and endothelial cells and aged mice (84 weeks old). Therapeutic effects of MSC-derived EVs were also found in cutaneous photoaging. Wu et al. (2021b) found that exosomes derived from human umbilical cord MSCs (hucMSC-ex) relieve ultraviolet (UV) radiation-induced DNA damage, inflammation, and apoptosis in HaCaT cells by trafficking 14-3-3ζ protein and upregulating the sirtuin 1 (SIRT1) levels. Cao et al. (2021) found that EVs from adipose-derived stem cells (ADSCs-EVs) ameliorate photoaging efficiently and safely. The topical treatment of ADSC-EVs reduces wrinkles, improves the collagen structure and density, and contributes to the clearance of inflammation.

**FIGURE 1:**
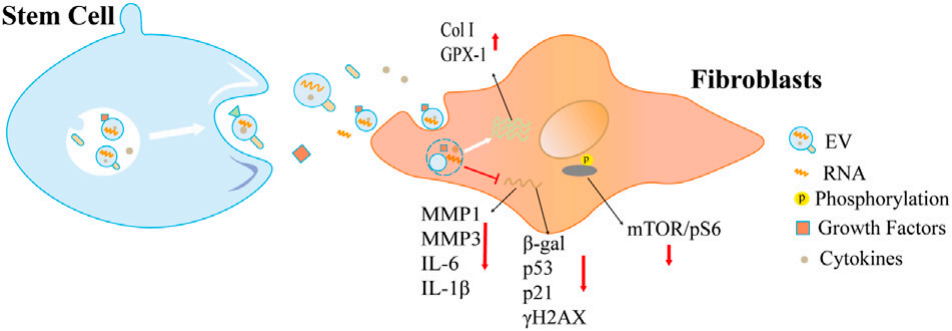
EVs derived from stem cells alleviate cellular aging. These EVs regulate the expression of aging/senescent markers, including p53, p21, γH2AX, *ß*-gal, and other senescence-associated secretory phenotype (SASP) factors, and efficiently improve the function of senescent cells through multiple mechanisms.

**FIGURE 2:**
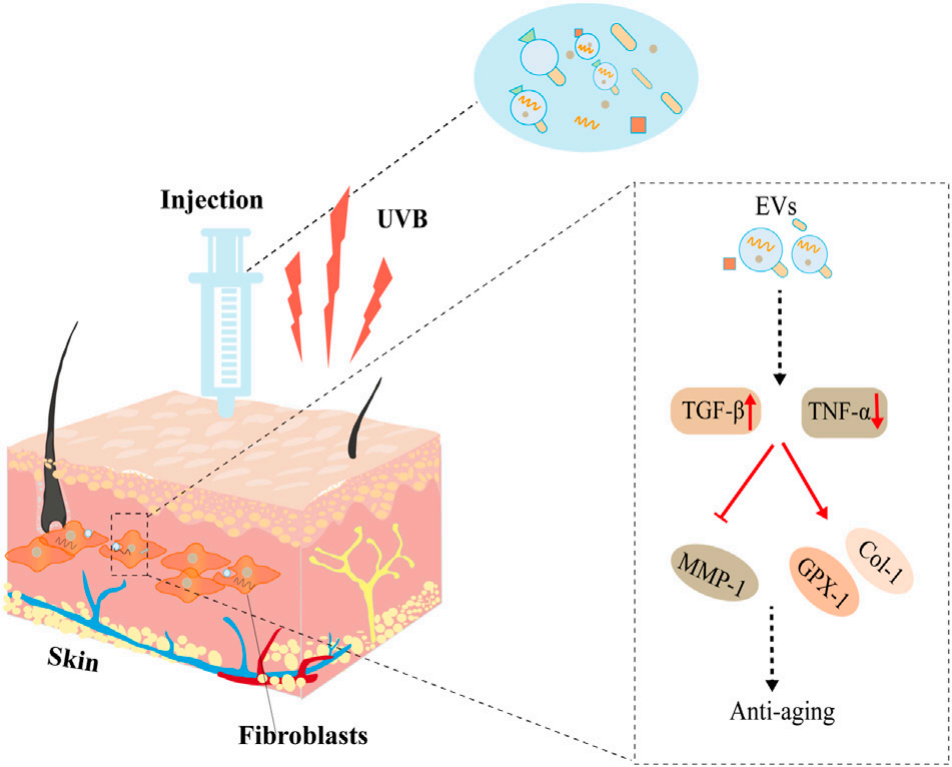
EVs derived from fibroblasts protect against UVB-induced senescence. After injection, EVs stimulate antioxidant activity (GPX-1), promote extracellular matrix reconstruction (TGF-β, Col-1, MMP1), improve cellular necrosis (TNF-α), and thus protect against UVB-induced aging.

Human pluripotent stem cell-derived exosomes (iPSC-exos) have been studied in the treatment of skin aging (Wu et al., 2021c). Oh et al. (2018) found that iPSC-exos ameliorated genotypic and phenotypic variations of photoaging human dermal fibroblasts. They also found that iPSC-exos reduces the expression of senescence marker (β-gal, matrix metalloproteinase 1 and 3) (MMP1 and MMP-3) and restores the secretion of collagen type I (Col-1) in both UV-induced and natural senescent dermal fibroblasts (Figure 1). In addition to stem cells, other EV sources have also been studied to optimizing the treatment of skin aging (Lee et al., 2021), such as skin fibroblast (Kim et al., 2017). Human dermal fibroblasts (HDFs) are one of the major cell types in the dermis, and their capacity in matrix regeneration gradually declines with age (Krebs et al., 2018). It has been reported that the exosomes derived from 3D-cultured HDF significantly improve the senescent phonotypes of dermal fibroblasts and nude mice (Hu et al., 2019). They regulate the expressions of tumor necrosis factor-alpha (TNF-α), MMP-1, transforming growth factor beta (TGF-β), and pro-Col-1 and thus promote collagen synthesis and improve aging phonotype. Furthermore, Deng et al. (2020) have shown that dermal fibroblast-derived EVs improve photoaging induced by ultraviolet radiation B (UVB) radiation. Pretreatment with these EVs significantly increased the expressions of glutathione peroxidase 1 (GPX-1) and extracellular matrix protein Col-1 and decreased the expression of MMP-1, thereby upregulating antioxidant activity and protecting dermal fibroblasts (Figure 2).

### EVs improve wound healing

About 2% of the populations of industrialized nations sustain non-healing wounds every year (Welt et al., 2009). For example, skin chronic wounds affect more than 6.5 million people in the United States (Sen et al., 2009). The incidence rate of chronic wounds significantly increases in aging populations, especially those who suffer from diabetes and obesity (Rodrigues et al., 2019).

**FIGURE 3:**
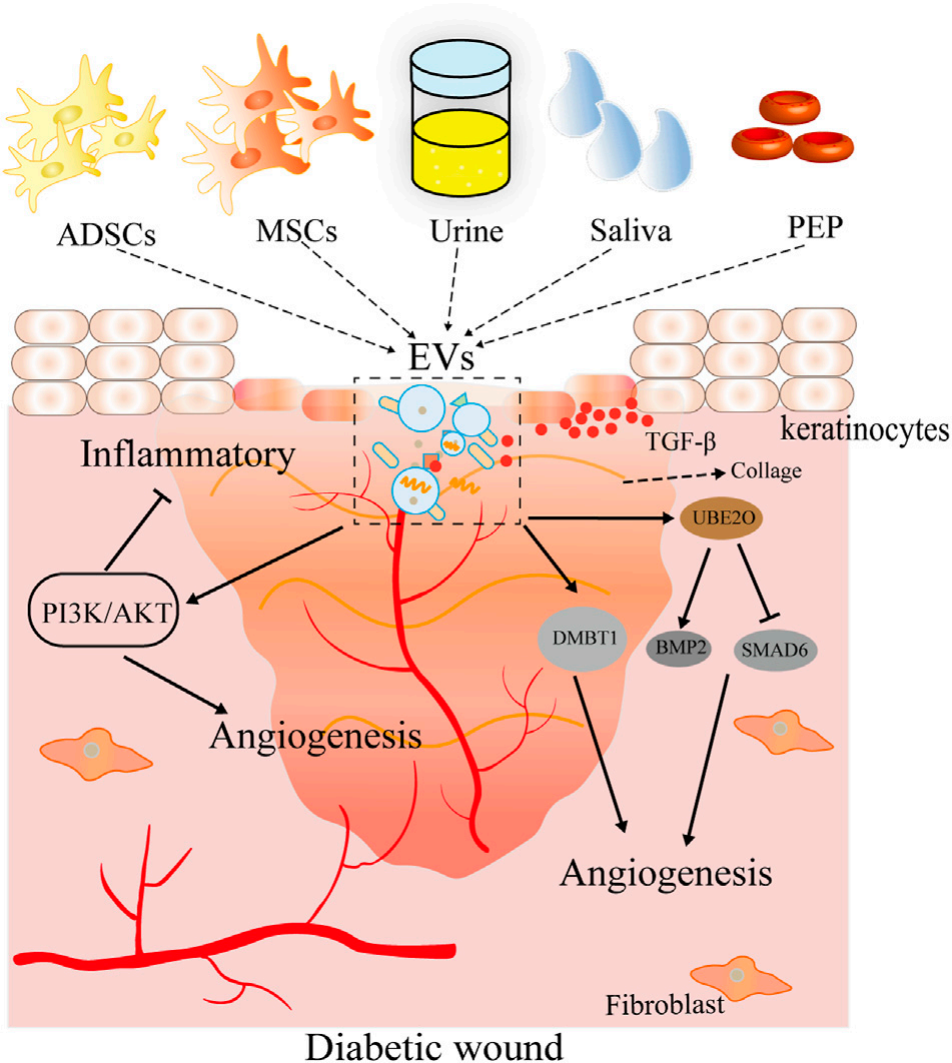
Therapeutic effects of EVs derived from multiple sources on skin wound healing. EV sources include pretreatment mesenchymal stem cells (hypoxia and pioglitazone) and body fluids (urine, saliva, and platelets). These EVs may promote viability, proliferation, and angiogenesis in cells and accelerate wound healing *in vivo*.

EVs, as a cell-free regenerative platform, show a strong potential in trauma treatment and wound management (Ji et al., 2021). Exosomes derived from hypoxic adipose stem cells regulate proliferation, migration, and extracellular matrix (ECM) metabolism of human skin fibroblasts by activating the PI3K/Akt pathway and thus improve the healing rate and quality of diabetic wounds (Wang et al., 2021a). Hu et al. (2021) reported that exosomes derived from pioglitazone-pretreated MSCs improve cell viability, proliferation, and angiogenesis ability of human umbilical vein vascular endothelial cells (HUVECs) injured by high glucose through activating the PI3K/AKT/eNOS pathway, thereby promoting ECM remodeling, angiogenesis, and diabetic wound healing in rodents. Chen et al. (2018) noted that exosomes from human urine-derived stem cells (USC-Exos) may provide a potential therapeutic strategy for diabetic wound healing by promoting angiogenesis *via* transferring deleted in malignant brain tumors 1 (DMBT1) protein. Human saliva contains numerous proteins and growth factors, which make it an effective tool promoting tissue regeneration (Torres et al., 2017; Scott and Munkley, 2019). Mi et al. (2020) noted that saliva-EVs promote HUVEC migration, proliferation, and angiogenesis by regulating the expression level of ubiquitin-conjugating enzyme E2O (UBE2O), SMAD family member 6 (SMAD6), and bone morphogenetic protein 2 (BMP2) and further enhance wound healing *via* promotion of angiogenesis. Platelets are newfound secretory cells and are an important EV source (Eisinger et al., 2018). Shi et al. (2021a) found that platelet exosomes accelerate wound healing by delivering bioactive TGF-β to the wound bed (Figure 3).

### EVs in medical cosmetics

Medical cosmetics are drawing increasing attention thanks to the development of human aesthetics. For example, EVs are a powerful ingredient for the management of skin tone in cosmetology (Yamaguchi et al., 2006; Cardinali et al., 2008). When skin is irradiated, melanin production is highly increased in melanocytes. Biosynthesis of melanin pigments is the first line of cutaneous defense after ultraviolet light irradiation (Maddodi et al., 2012; Sun et al., 2020). However, after excessive and long UV irradiation, excessive melanin production inopportune metabolism and irregular accumulation in skin cells will induce skin burning, tanning, and pigmentation (Jeon et al., 2016). Among these, close attention has been given to skin pigmentation (Romano et al., 2021).

**FIGURE 4:**
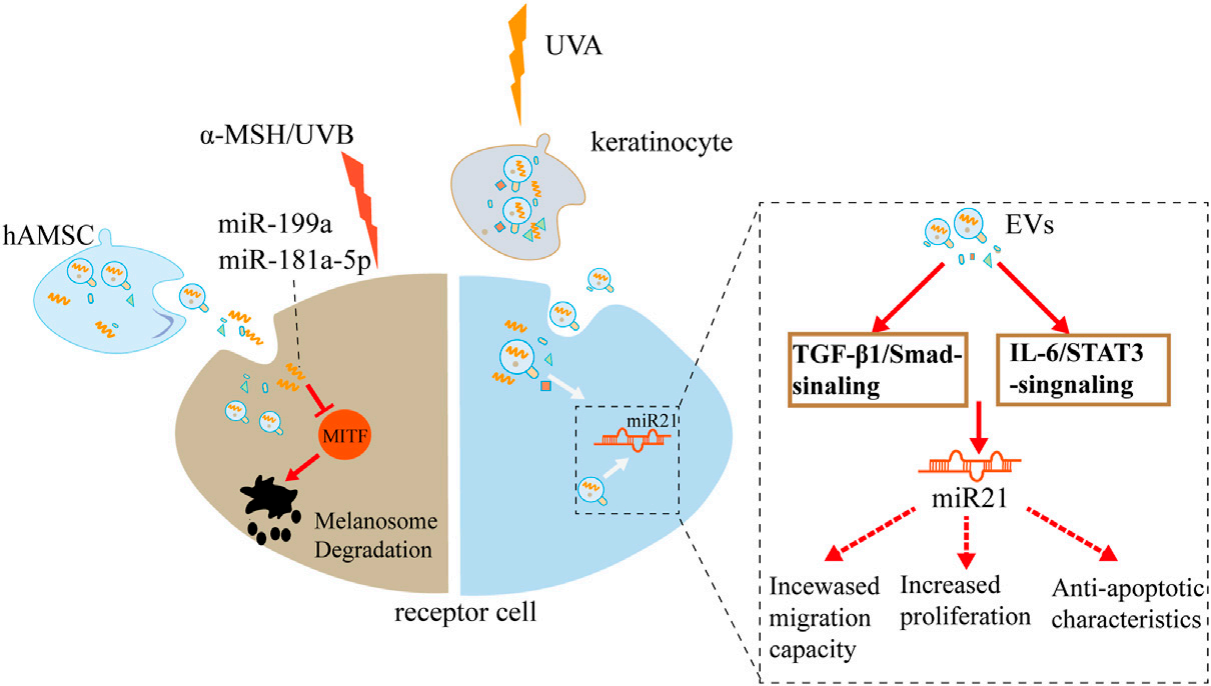
Potential effects of EVs on pigmentation: hAMSC-derived exosome and UVA-driven keratinocyte EVs remarkably suppress melanin synthesis, respectively, through multiple signaling pathways. hAMSC, human adipose mesenchymal stem cells; UVA, Ultraviolet Radiation A.

Improving pigmentation in the skin has been a long-term goal for cosmetic and medical applications. Wang et al. (2021b) demonstrated that human amniotic MSC-derived conditional medium and exosomal microRNA (miR-181a-5p and miR-199a) significantly suppress microphthalmia-associated transcription factor-dependent melanin synthesis and promote autophagy-based melanosome degradation in multiple aged models, including *a*-melanocyte-stimulating hormone-irritated B16F10 cells, UVB-treated human skin, and photo-aged mouse ears. Wäster et al. (2020) reported that Ultraviolet Radiation A (UVA) irradiation-stimulated keratinocyte EVs promote cell renewal and epidermis thickening, strengthening the function of skin barrier against sunlight exposure through a complex gene regulatory circuit. These genes involve extracellular regulated protein kinases (ERK), c-Jun N-terminal kinase (JNK), programmed cell death factor 4 (PDCD4), phosphatase and tensin homology deleted on chromosome 10 (PTEN), TGF-β, interleukin-6 (IL-6)/signal transducer and activator of transcription 3 (STAT3), and microRNA 21 (Figure 4).

### Engineered EVs for skin repairs

While EVs are used in a therapeutic role for various diseases and injuries, they are far from realizing their full potential in drug delivery or regenerative treatment (Burgos-Ravanal et al., 2021). As research continues, a number of modifications have been adopted to optimize the targeting and efficiency of EV therapy (Ural et al., 2021). Here, we summarize the recent advances of engineered and combination therapy of EVs for skin repairs.

#### Hydrogels

Hydrogels are three-dimensional nanofiber materials that are made up of physically or chemically cross-linked hydrophilic polymer networks in structure. They are special materials that swell and retain a substantial quantity of water and keep their constrained integrity in structure and dimension (Chai et al., 2017).

Hydrogels have been widely used in many biomedical areas, including cell therapy, drug delivery, biosensing, and tissue engineering (Shah et al., 2019). Encapsulating EVs in hydrogels can keep their biological activity and give a controlled release. Wang et al. (2019a) developed a self-healing multifunctional FHE hydrogel, which is composed of Pluronic F127, oxidative hyaluronic acid, and Poly-ε-L-lysine (EPL). The FHE hydrogel performs excellently in the delivery and release of bioactive substance (Tan et al., 2013). The authors demonstrated that when compared with single treatment using exosomes or FHE hydrogel alone, the combined therapy (hydrogel-encapsulated exosomes) stimulated the proliferation, migration, and tube formation ability of HUVECs and significantly enhanced wound closure rates, angiogenesis, collagen deposition, and re-epithelization of diabetic wounds.

Hypoxia, ischemia, oxidative stress, and infection are critical clinical hallmarks of non-healing chronic diabetic wounds. Manganese dioxide (MnO_2_) nanosheets are capable of detoxification of endogenous ROS (He et al., 2018). To accelerate diabetic wound repair, Xiong et al. (2022) introduced a self-healing, injectable, and adhesive hydrogel, which is mixed with MnO_2_, fibroblast growth factor 2 (FGF-2) and exosomes. They demonstrated that this “All-in-One” hydrogel is able to form an antibacterial layer that covers the wound, improves the survival and function of human skin fibroblast and HUVEC, and facilitates the healing of diabetic wounds. Antibacterial activity, alleviating oxidative stress, restoring O_2_ supply, and releasing exosomal miR-223 and FGF-2 were considered to be the underlying mechanisms. Shiekh et al. (2020) introduced an oxygen-releasing material combining antioxidant polyurethane with ADSCs-EVs (OxOBand). Their results indicated that OxOBand promoted wound closure, collagen deposition, epithelial regeneration, and angiogenesis and decreased oxidative stress in diabetic wounds.

Chitosan hydrogels (CSs) have good thermal sensitivity and loose porous structural properties. Zhao et al. (2021) suggested that CS hydrogel-encapsulated EVs improved skin aging by enhancing the function of aged dermal fibroblasts. They found that CS hydrogel-incorporated EVs (CS-EVs) target the dermal fibroblasts with replicative senescence, promote the proliferation of aged cells, enhance the synthesis of ECM proteins, and inhibit the upregulation of MMPs *in vitro*. After multi-site subcutaneous injection of CS-EVs, the treated skin of aged mice is manifested as the reduced expression of SASP-related factors, the increased expression of collagen, and the restoration of tissue structures.

**FIGURE 5:**
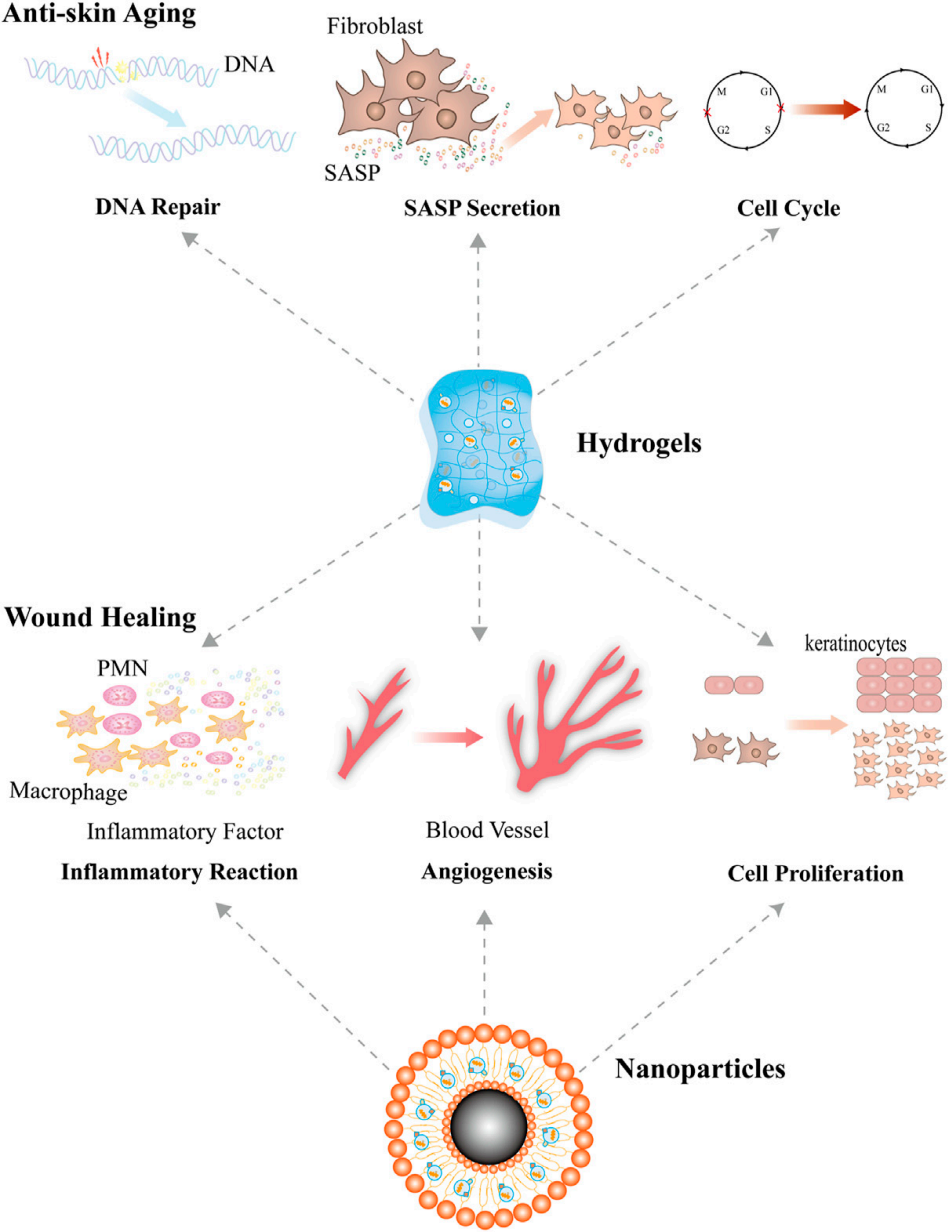
Engineered EVs for skin repairs. Combining EVs with hydrogels or nanoparticles significantly optimizes their therapeutic effects. Compared with natural EVs, engineered EVs have stronger effects in promoting cell proliferation, accelerating DNA repair, enhancing angiogenesis, modulating inflammation, and suppressing the secretion of SASP factors, and therefore they have better performance in the therapy of chronic wounds.

Hyaluronic acid hydrogels (HA-Gels) have the potential to reduce wrinkles by physically filling but not biologically stimulating collagen generation. Recently, You et al. (2021) published a potential dermal filler using stem cell-derived EV-bearing HA-Gels (EV-HA-Gels). They demonstrated that EV-HA-Gels induced the overexpression of CD301b on macrophages. Some miRNAs, such as let-7b-5p and miR-24-3p, participated in the effects of EV-HA-Gels by improving the proliferation of fibroblasts in the dermis region. *In vivo* experiments indicated that EV-HA-Gels significantly stimulated collagen synthesis in treated dermis, which is 2.4-fold higher than that in HA-Gel-treated dermis. These increased collagens were maintained for at least 24 weeks in the dermis (Figure 5).

#### Nanoparticles

Magnetic nanoparticles (NPs) are a kind of nanomaterial that are characterized by controlled targeting, easy synthesis, low toxicity, and good biocompatibility (Hohnholt et al., 2011). Recent studies have reported several strategies of target delivery that combine EVs with magnetic NPs (Qi et al., 2016; Jia et al., 2018). Wu et al. (2020) fabricated novel exosomes named as mag-MSC-Exos. These exosomes are derived from bone MSCs precultured with Fe_3_O_4_ NPs and a static magnetic field. The authors found that when compared with cells treated with control MSC-Exo, other cells treated with mag-MSC-Exo showed better performance in proliferation, migration, and angiogenesis *in vitro*, and mag-MSC-Exos transplanted locally into rat skin wounds can accelerate wound closure, reduce scar area, and enhance angiogenesis *in vivo*. In addition, Li et al. (2020) demonstrated that the exosomes that are derived from NPs-loaded MSCs have strong potential in the therapy of cutaneous wound *in vivo* by increasing the targeting delivery efficiency of MSC-Exos (Figure 5).

#### Other modifications

Liposomes are a kind of tiny lipid vesicles that are surrounded by a membrane bilayer that is mainly composed of phospholipids and cholesterol, which can entrap drugs and be used as valuable drug delivery systems (Hu and Zhang, 2012). However, their characteristics of non-targeted, low efficacy and short half-life have prevented the translational application of liposomes (Gibis et al., 2013; Pattni et al., 2015). Evers et al. (2022) proposed a kind of EV-liposome hybrid NPs. They demonstrated that hybrid NPs containing target genes and EV-surface makers enable the targeted delivery of genes to specific cells.

Several approaches have been used to enrich specific inclusions in EV cargo (Banerjee, 2011). Li et al. (2018) demonstrated that when compared to standard MSC-EVs, EVs derived from nuclear factor-E2-related factor 2 (NRF2) overexpressing MSCs packaged more NRF2 and showed enhanced improvement in inflammation and oxidative stress and wound healing. Inducing the direct link between target gene and EV membrane component is another direction that has been exploited (Lan et al., 2018). A light-based reversible linker has been used to attach a specific protein to CD9, which is a membrane protein on the surface of EVs (Zou et al., 2018).

Recently, Kojima et al. (2018) reported a novel and feasible method for packaging specific mRNA into EVs, the EXOsomal transfer into cells (EXOtic) system (Ausländer et al., 2012). In this system, the archaeal ribosomal peptide L7Ae is fused to EV marker protein CD63, which allows for recruitment and encapsulation of those mRNAs containing C/Dbox to budding exosomes. The C/Dbox sequence was cloned at the 3′end to express mRNA containing the C/Dbox RNA domain.

### Limitations and perspectives

While significant advances have been made in recent years, there are still some limitations that have suppressed the translational application of EVs. First, variations in EV isolation methods complicate reproducibility and comparative study. Although a growing number of techniques and methods have been developed based on the minimal requirements initiated by the International Society for Extracellular Vesicles (MISEV 2018), it is worth noting that different isolation techniques or even different parameters will cause great diversity in the characteristic and package contents of EVs (Gurunathan et al., 2019). Without a well-accepted and golden standard protocol to standardize the procedure of EV isolation and quality control, each laboratory or company will naturally establish its own “EVs” (Théry et al., 2018). A series of field-consensus, rigorous, and type-specific protocols for isolation and quality control of EVs are urgently required.

Second, the output of EVs is too low to perform a large-scale study in large animals or patients. On one hand, novel bioreactors have been designed to enlarge the output of EVs. Published data have shown that bioreactors can produce approximately 5- to 10-fold output than routine cultures using cell factories and T-flasks (Gimona et al., 2017). On the other hand, mammalian cells produce only a few EVs in natural condition. Therefore, molecular strategies have been investigated to increase the production of EVs through the overexpression of core proteins regulating EV biogenesis (Kojima et al., 2018; Ortega et al., 2019). Modifications of the cell culture conditions may further improve the output of EVs, such as biomaterial coating, extracellular matrix composition, interface pattern and rigidity, cell metabolism, and other physical and chemical stimulations.

Third, there is insufficient validation about the safety and efficacy of EV therapy in humans. Contaminations and residues brought by the supplement reagents of cell culture (e.g., serum and other serum replacement) are inevitable and difficult to wash out. To reduce their influence, ultracentrifugation and ultrafiltration have been adopted before supplement usage. While earlier research has suggested that the allogeneic EVs have extreme low immunogenicity, recent studies have revealed that EVs secreted by virus-infected cells or immune cells are immunogenic and can deliver cell-specific molecules over long distances to elicit immune responses (Hong et al., 2021). Some EV sources, such as MSCs, are known to be lowly immunogenic, and therefore EVs derived from them are expected not to be immunogenic; however, there is little evidence for this at the moment (Zheng et al., 2018). To allow their use in humans and avoid side effects or function loss, the immunogenicity of EVs have to be tested.

Finally, the majority of EV studies are limited to rodent models, and most of the main models for the detection of the therapeutic effect of EVs are focused on 2D culture systems and animal models (Fonseca et al., 2017). These approaches and other more advanced skin experimental models (e.g., tissue biopsy, 3D cell studies, and 3D bioprinting) all have drawbacks (e.g., lacking cell-cell/-matrix crosstalk and natural mechanical and chemical cues). To overcome these limitations, skin-on-a-chip (SoC) biomimetic artificial skin models have been developed. SoC can impart a fine control over the microenvironment, induce some mechanical cues, help analyze the features of normal and diseased human skin, and develop and test substances for pharmaceutical and cosmetic applications. In brief, SoC has significant potential during the analysis of the effect of EV therapy (Sutterby et al., 2020).

EVs can carry proteins, nucleic acids, lipids, and metabolites. Although studies of EVs in skin disease are still in the early stages, mainly focusing on proteins and nucleic acids, they have shown promising results in a number of clinical trials and applications. Other compositions in EVs are poorly investigated, including lipids and metabolites. We would expect that they need to be further investigated in the future, both biologically and clinically. We have summarized the features of EVs in Table 1.

**TABLE 1:** Features of EVs in promoting wound healing, anti-aging, and anti-pigmentation.

Cell type	Effect	Source of EVs/exosomes	Evidence and clues	Features		References
				Advantages	Disadvantages	
Stem cells	Improving skin aging	BM-MSCs-EVs	↓P16, P21, IL-6 and IL-1β	High safety, low risk of rejection, better biocompatibility, being readily storage, and few tumorigenic and functional diversification	High costs and tedious sample preparation, and potential ethical issues	(Oh et al. (2018); Hu et al. (2019); Wang et al. (2021a); Shi et al. (2021b); Wang et al. (2021b); Wu et al. (2021b); Dorronsoro et al. (2021); Hu et al. (2021)
			↑health span			
	Improving skin aging	GMSC-EVs	↓β-gal, P53, P21, γH2AX & mTOR/P53			
			↑ cell proliferation			
	Improving skin aging	hucMSC-exo	↑autophagy			
			↓DNA damage, inflammation and oxidative stress			
	Improving skin aging	iPSC-exos	↑Col-1			
			↓β-gal, MMP-1 and MMP-3			
	Anti-pigmentation	hASCs-evs	↓melanin synthesis			
			↑autophagy-based melanosome degradation			
	Improving wound healing	ADSCs-exo	↑proliferation, collagen metabolism and migration			
			↑healing rate and quality of diabetic wound			
	Improving wound healing	PGZ-Exos	activating the PI3K/AKT/eNOS pathway			
			↑cell viability, proliferation, and angiogenesis ability			
			↑ECM remodeling and diabetic wound healing			
Adult cells	Improving skin aging	3D HDF-XOs	↓TNF-α, MMP-1	High safety, low risk of rejection, better biocompatibility, and easy accessibility	Single function and low yield	Hu et al. (2019); Deng et al. (2020); Wäster et al. (2020)
			↑TGF-β & pro-Col-1			
	Improving skin aging	Fb-EVs	↑GPX-1, Col-1 &antioxidant activity			
			↓MMP-1			
	Anti-pigmentation	Ultraviolet Radiation A (UVA) irradiation-stimulated keratinocyte EVs	↑cell renewal, epidermis thickening and anti-apoptosis response			
Others	Improving wound healing	USC-Exos	transferring DMBT1	Useful for noninvasive early diagnosis, easy accessibility, and more closely related to disease and lesion tissue	Samples prone to contamination and relatively large risk for the operators	Chen et al. (2018); Mi et al. (2020); Shi et al. (2021a)
			↑angiogenesis			
			↑diabetic wound healing			
	Improving wound healing	saliva-Exos	↓SMAD6			
			↑ BMP2, angiogenesis and wound healing			
	Improving wound healing	platelets exosomes	↑angiogenesis			
			↑wound healing			
Hydrogels	Improving wound healing	FHE hydrogel	↑proliferation, migration and tube formation ability of HUVECs, wound closure rates, angiogenesis, collagen deposition and re-epithelization	Controlled cost and easy to prepare beforehand	Single function, poor biocompatibility, and heterogeneity in material property	(Wang et al. (2019a); Shiekh et al. (2020); You et al. (2021); Zhao et al. (2021); Xiong et al. (2022)
	Improving wound healing	The manganese dioxide (MnO_2_) nanosheets	↑ the survival and function of human skin fibroblast and HUVEC & the healing of diabetic wounds			
	Improving wound healing	OxOBand	↑wound closure, collagen deposition, epithelial regeneration and angiogenesis			
			↓oxidative stress in diabetic wounds			
	Improving wound healing	CS	↑ the proliferation of aged cells and the synthesis of ECM proteins			
			↓ the upregulation of MMPs			
	Improving wound healing	HA-Gels	↑the proliferation of fibroblasts and the collagen synthesis			
			↓wrinkles			
Nanoparticles	Improving wound healing	mag-MSC-Exos	↑wound closure and angiogenesis	Controlled targeting, easy synthesis, strong function, and personalized modification	Poor biocompatibility, poor safety, and uneven distribution	Wu et al. (2020; Wu et al. (2021a)
	Improving wound healing	Exosomes derived from NPs-loaded MSCs	↑wound closure and angiogenesis			

Abbreviations: bone marrow-derived mesenchymal stem cell-extracellular vesicles, BM-MSCs; gingiva-derived mesenchymal stem cell-extracellular vesicles, GMSC-EVs; exosomes derived from human umbilical cord MSCs, hucMSC-exo; human pluripotent stem cell-derived exosomes, iPSC-exos; extracellular vesicles derived from human amniotic stem cells, hASCs-evs; Exosomes of adipose stem cells, ADSCs-exo; exosomes derived from three-dimensional spheroids, 3D HDF-XOs; extracellular vesicles derived from fibroblasts, Fb-EVs; Ultraviolet Radiation A, UVA; exosomes derived from MSCs, pretreated with pioglitazone, PGZ-Exos; exosomes from human urine-derived stem cells, USC-Exos; saliva-derived exosomes, saliva-exos; composition of pluronic F127, oxidative hyaluronic acid, and Poly-ε-L-lysine (EPL), FHE, hydrogel; material combining antioxidant polyurethane with ADSCs-EVs, OxOBand; chitosan hydrogels, CS; hyaluronic acid hydrogels, HA-Gels; exosomes are derived from bone MSCs, precultured with Fe3O4 NPs, and a static magnetic field, mag-MSC-exos; nanoparticles,NPs; interleukin-6, IL-6; metalloproteinase 1, MMP-1; metalloproteinase 3, MMP-3; *ß*-galactosidase, *ß*-gal; mammalian target of rapamycin, mTOR; collagen type I, Col-1; tumor necrosis factor-alpha, TNF-α; transforming growth factor beta, TGF-β; glutathione peroxidase 1, GPX-1; deleted in malignant brain tumors 1, DMBT1; SMAD, family member 6, SMAD6; bone morphogenetic protein 2, BMP2; extracellular matrix, ECM.

The majority of EV studies is basic research and are rarely reported in commercial and industrial products. Only a few major product brands have developed EV-related products, such as Condensation E.R.T, Oyon-Young, and ASCE. Even so, the potential of EVs in skin care has attracted worldwide attention. A series of problems (e.g., preparation, storage, and preservation of life of EVs) have prevented EVs from being widely promoted to reach an acceptable price level. In addition, EVs have highly complex contents that act in a cooperative manner, and little evidence is available to describe the dominant player in the EV component. With the rapid development of science and technology, we believe that the component-specific and larger-scale production of EVs will be realized in the coming future.

## Conclusion

In this article we have summarized the current development of the therapeutic potentials of EVs in skin aging, refractory wound and pigmented dermatosis, and have reviewed a series of novel methods for improving the efficacy of EV therapy.
